# Supplementary material for: 7-Phenylheptanoic Acid-Hydroxypropyl β-Cyclodextrin Complex Slows the Progression of Renal Failure in Adenine-Induced Chronic Kidney Disease Mice
Source: Toxins (Basel). 2024 Jul 12;16(7):316. doi: 10.3390/toxins16070316 (PMC11281668; doi:10.3390/toxins16070316)
Supplement: Supplementary file 1 [file toxins-16-00316-s001.zip › toxins-3045770-supplementary.pdf]

7-Phenylheptanoic Acid-Hydroxypropyl β-Cyclodextrin Complex Slows the Progression of Renal Failure in Adenine-Induced Chronic Kidney Disease Mice

Kindness Lomotey Commey, Airi Enaka, Ryota Nakamura, Asami Yamamoto, Kenji Tsukigawa, Koji Nishi, Masaki Otagiri and Keishi Yamasaki

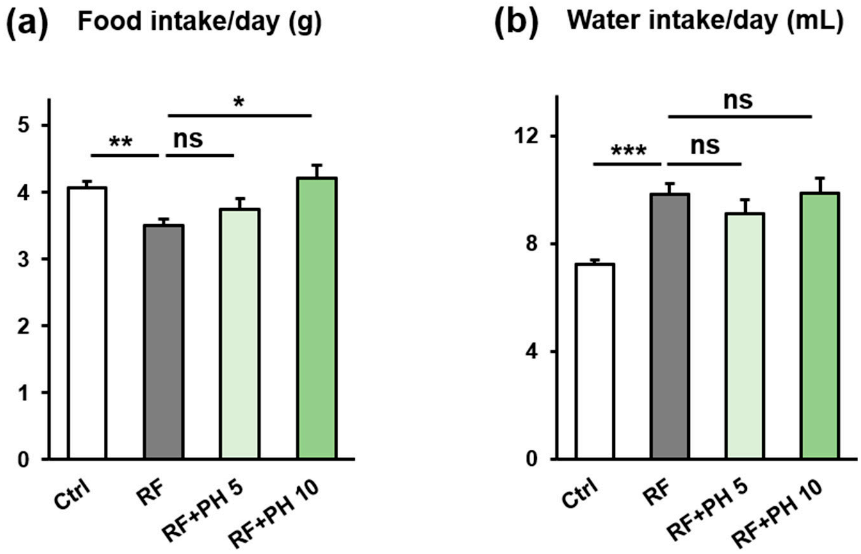

Figure S1. (**a**) Food intake and (**b**) Water intake of adenine-induced CKD mice receiving a prophylactic supplementation of PH-HPβCD. The experimental protocol is described in Section 5.4.2. Values expressed as mean ± SE (n = 4–5). One-way ANOVA, followed by Bonferroni correction. * $p < 0.05$, ** $p < 0.01$, *** $p < 0.001$, ns, no significant difference.

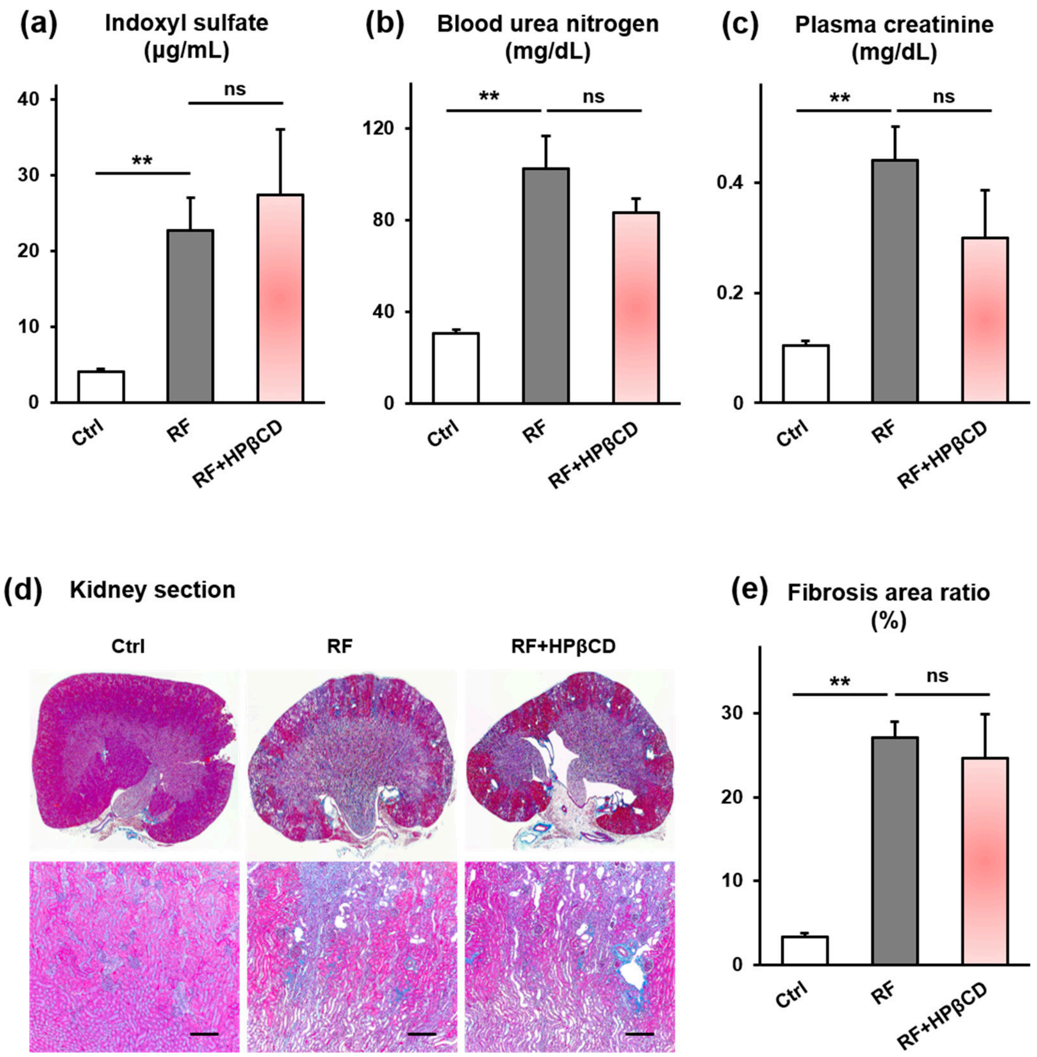

Figure S2. Effect of prophylactic supplementation with HPβCD alone on the renal function of adenine-induced CKD mice. (**a**) Plasma IS. (**b**) BUN. (**c**) Plasma CRE. (**d**) Representative micrographs of Masson's trichrome-stained kidney sections; scale bar, 200 μm. (**e**) Quantitative analysis of tubular fibrosis area. The experimental protocol was identical to that used in Section 5.4.2., however, the adenine feed was supplemented with HPβCD alone in place of PH-HPβCD. The dose of HPβCD based on food intake was 48.62±2.04 mg/kg/day. Values expressed as mean ± SE (n = 4–5). One-way ANOVA, followed by Bonferroni correction. ** $p < 0.01$, ns, no significant difference.

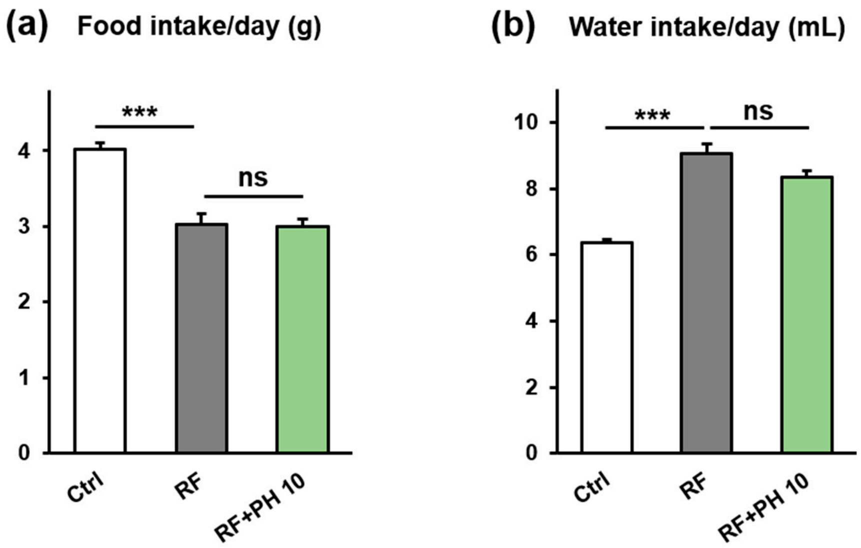

Figure S3. (**a**) Food intake and (**b**) Water intake of moderately advanced adenine-induced CKD mice treated with PH-HPβCD. The experimental protocol is described in Section 5.4.3. Values expressed as mean ± SE (n = 4–5). One-way ANOVA, followed by Bonferroni correction. *** $p < 0.001$, ns, no significant difference.

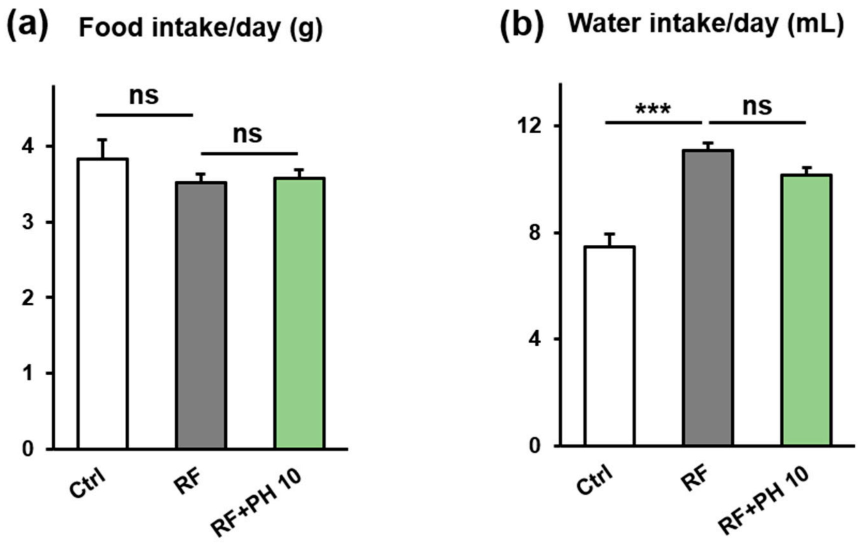

Figure S4. (**a**) Food intake and (**b**) Water intake of 5/6 nephrectomized mice treated with PH-HPβCD. The experimental protocol is described in Section 5.4.4. Values expressed as mean ± SE (n = 4–5). One-way ANOVA, followed by Bonferroni correction. *** $p < 0.001$, ns, no significant difference.

PH has no Inhibitory Effect on Sulfotransferase (SULT) Activity In Vitro

The effect of PH on sulfonation of 3-hydroxyindole to indoxyl sulfate (IS) by sulfotransferase enzyme was investigated in vitro. The assay was conducted as previously reported [33]. The reaction mixture contained 10.6 mM dithiothreitol (Sigma-Aldrich Co., St. Louis, MO, USA), 0.2 mM 3'-phosphoadenosine 5'-phosphosulfate (PAPS) (Sigma), 3.3 units porcine liver esterase (Sigma), and 12 μM of indoxyl acetate in acetone (acetone < 5% v/v of the reaction mixture) in 23.3 mM potassium phosphate buffer (pH 7.0). Following a 2 min incubation to generate 3-hydroxyindole in situ from the indoxyl acetate, increasing known concentrations of PH in 23.3 mM potassium phosphate buffer (pH 7.0) was added. The sulfonation reaction was then initiated by adding human liver cytosol (0.1 mg protein) (Sigma). After incubation for 10 min at 37°C, the reaction was terminated by adding ice-cold methanol and then centrifuged at 12000× *g* for 2 min. The content of IS in the supernatant was determined by HPLC (as described in Section 5.4.6.).

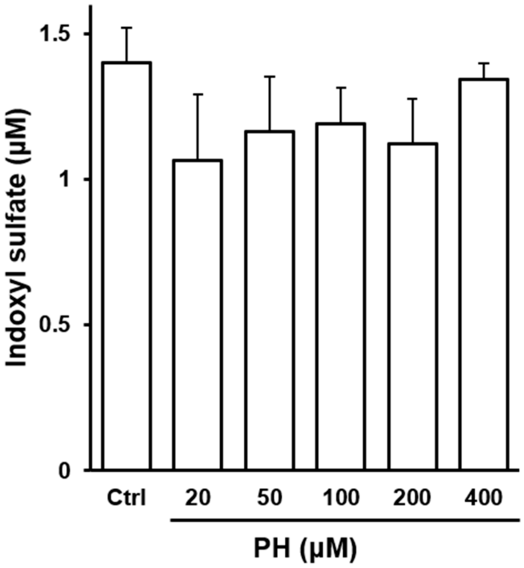

Figure S5. Effect of PH on SULT activity in vitro. Sulfonation of 3-hydroxyindole to IS by SULT was not affected by the presence of PH.

Reference

33. Banoglu, E.; King, R.S. Sulfation of indoxyl by human and rat aryl (phenol) sulfotransferases to form indoxyl sulfate. *Eur. J. Drug Metab. Pharmacokinet.* **2002**, *27*, 135–140. https://doi.org/10.1007/BF03190428.
